# Using Social Media to Characterize Public Sentiment Toward Medical Interventions Commonly Used for Cancer Screening: An Observational Study

**DOI:** 10.2196/jmir.7485

**Published:** 2017-06-07

**Authors:** Omar Metwally, Seth Blumberg, Uri Ladabaum, Sidhartha R Sinha

**Affiliations:** ^1^ Department of Clinical Informatics University of California, San Francisco San Francisco, CA United States; ^2^ Department of Internal Medicine Highland General Hospital Oakland, CA United States; ^3^ Francis I. Proctor Foundation University of California, San Francisco San Francisco, CA United States; ^4^ St Mary's Medical Center San Francisco, CA United States; ^5^ School of Medicine, Division of Gastroenterology Stanford University Stanford, CA United States

**Keywords:** Twitter, sentiment analysis, cancer screening, colonoscopy, mammography, Pap smear, Papanicolaou test, social media, early detection of cancer

## Abstract

**Background:**

Although cancer screening reduces morbidity and mortality, millions of people worldwide remain unscreened. Social media provide a unique platform to understand public sentiment toward tools that are commonly used for cancer screening.

**Objective:**

The objective of our study was to examine public sentiment toward colonoscopy, mammography, and Pap smear and how this sentiment spreads by analyzing discourse on Twitter.

**Methods:**

In this observational study, we classified 32,847 tweets (online postings on Twitter) related to colonoscopy, mammography, or Pap smears using a naive Bayes algorithm as containing positive, negative, or neutral sentiment. Additionally, we characterized the spread of sentiment on Twitter using an established model to study contagion.

**Results:**

Colonoscopy-related tweets were more likely to express negative than positive sentiment (negative to positive ratio 1.65, 95% CI 1.51-1.80, *P*<.001), in contrast to the more positive sentiment expressed regarding mammography (negative to positive ratio 0.43, 95% CI 0.39-0.47, *P*<.001). The proportions of negative versus positive tweets about Pap smear were not significantly different (negative to positive ratio 0.95, 95% CI 0.87-1.04, *P*=.18). Positive and negative tweets tended to share lexical features across screening modalities. Positive tweets expressed resonance with the benefits of early detection. Fear and pain were the principal lexical features seen in negative tweets. Negative sentiment for colonoscopy and mammography spread more than positive sentiment; no correlation with sentiment and spread was seen for Pap smear.

**Conclusions:**

Analysis of social media data provides a unique, quantitative framework to better understand the public’s perception of medical interventions that are commonly used for cancer screening. Given the growing use of social media, public health interventions to improve cancer screening should use the health perceptions of the population as expressed in social network postings about tests that are frequently used for cancer screening, as well as other people they may influence with such postings.

## Introduction

The US Preventive Services Task Force and numerous professional societies endorse colonoscopy, mammography, and Pap smear as effective screening modalities for colon, breast, and cervical cancer, respectively. Over 350,000 cases of these cancers are diagnosed yearly in the United States [1-5]. Despite the effectiveness of these and other screening modalities in reducing cancer-related mortality, millions of Americans do not undergo screening [6-13]. The reasons for this lack of adherence, particularly for these 3 tests, are multifold. Colonoscopy, mammography, and Pap smear are generally considered more invasive or involved than exclusively laboratory-based screening tests, such as those for high cholesterol or diabetes. Briefly, colonoscopy generally involves visualization via a flexible endoscope inserted into the rectum, and often biopsy, of colonic mucosa. To increase the chances of complete visualization of colonic mucosa, patients are typically advised to adhere to a restricted diet with avoidance of solid food the day prior to the procedure and are frequently required to drink large volumes of bowel-cleansing solutions that result in frequent bowel movements [14]. Mammography involves radiographic imaging after compression of the breast tissue, a process that can be uncomfortable for many women [15]. Pap smear entails insertion of a speculum into the vagina and use of a brush to scrape a sample of cervical cells, which allows the operator to examine cells microscopically for malignant and premalignant changes, but it can also be associated with pain and anxiety [16].

Patient questionnaires have identified discomfort, embarrassment, and various socioeconomic factors as barriers to participation in cancer screening [4,17]. However, studying patient perceptions of modalities commonly used for cancer screening using formal surveys is limited by several factors. First, these surveys can be costly to administer and do not provide real-time actionable information [18]. Second, monitoring the spread and changes in sentiment over time is limited by cost and diminishing response rates. Third, surveys ask specific questions and typically provide limited possible responses, which qualitatively and quantitatively limits the range of data generated by these interventions [19]. Infodemiology, which includes exploration of the distribution and determinants of information on the Internet to improve public health, provides an alternative method to study societal perceptions of health care, such as their sentiment regarding commonly used cancer screening interventions [20]. Infodemiologic studies have investigated numerous aspects of health, including attitudes toward and spread of illness as expressed on social media, search engines, and blogs; sentiment in chronic diseases; and the effectiveness of smoking cessation campaigns [20-24]. Analysis of social media postings offers a unique opportunity to overcome the limitations of conventional surveys and to understand core health care issues, such as why screening recommendations are often not followed. Among such networks, Twitter is relatively unique in that vast amounts of data are publicly available. Revealing differences in sentiment on social media toward various tools commonly used for screening and analyzing how interventions to improve screening affect perception may lead to understanding how screening adherence can be increased.

In this study, we applied established methods in sentiment analysis and machine learning to Twitter data to characterize sentiment toward common interventions used for cancer screening. Similar methods have been applied to characterize patient attitudes toward various medical topics, including vaccines, illness, pain, and drug use [19,25-29]. We also quantified the way in which sentiment regarding interventions commonly used for cancer screening spreads on social networks, offering a unique opportunity to both understand health-related discourse propagation and gain insight on how to engineer outreach efforts more effectively.

## Methods

### Recruitment

We used the Twitter (Twitter, Inc) search application programming interface (API) to collect over 30,000 English-language tweets relating to colonoscopy (10,262), mammography (12,002), and Pap smear (10,583) [12]. All tweets were collected on consecutive days over a 6-week period from December 2015 to January 2016. Colonoscopy-related tweets were identified by querying for the term “colonoscopy.” Mammography-related tweets were identified by querying for the term “mammogram” or “mammography.” Pap smear-related tweets were identified by querying for “pap smear,” “pap test,” “Papanicolaou test,” “Papanicolaou AND screening,” “pap AND cervical cancer,” “pap AND pelvic exam,” or “pap AND HPV.” We obtained the data set by writing code to manually collect these publicly available tweets through the Twitter API, which is a sampling of up to 1% of the total number of tweets at any time (ie, the Twitter Firehose) [30-32]. The question of how representative the Twitter API is of the Firehose has been rigorously studied, and the limitations are discussed in the “Limitations” section below. Briefly, the quantity and quality of tweets delivered via the API depends on the keywords used to query the tweets, the user IDs specified, and geographic tags (if present). The Twitter API begins sampling using an undisclosed method once the 1% tweet threshold is reached. Following the precedent set by other Twitter-based studies, and according to our institutional review board’s recommendations to exempt this study from review, we did not obtain consent from Twitter users, since we used the data in aggregate, and these data are publicly available. For each tweet, we recorded the content of the message and the number of retweets (how many times the tweet was propagated by other users).

### Sentiment Classification

We classified sentiment in 2 separate steps as described in the literature [33,34]. First, 1500 tweets (500 for each screening modality) were manually labeled by an investigator as containing positive, negative, or neutral sentiment and were used to train the classifiers. For each screening modality, we trained a naive Bayes classifier, a classification algorithm in which training is based on prior probabilities with different variables assumed to be independent of one another a priori, to categorize all tweets as carrying either positive or nonpositive sentiment [35]. Then, we trained a second naive Bayes classifier to categorize all tweets as carrying either a negative sentiment or a nonnegative sentiment. Tweets that were positive and nonnegative were ultimately labeled as positive. Tweets that were negative and nonpositive were ultimately labeled as negative. Tweets that were classified as both nonpositive and nonnegative were grouped into a third, neutral category. No tweets were classified as both positive and negative.

### Validity

To assess consistency in labeling, a random subset of the 1500 tweets were relabeled by the same investigator, with 96% concordance. To establish validity of our labeling system, a second investigator independently labeled tweets, with an interobserver concordance of 95%. We characterized the accuracy of the classification algorithm by using 1200 of the labeled tweets as a training set and the remaining 300 as a testing set [36]. Our decision to use 75% of labeled tweets for training and 25% of labeled tweets for testing is consistent with validity assessment common in the machine learning literature; 20% to 33% of a labeled set is commonly used for testing purposes, with the remaining dataset used for training [36]. We inferred the true proportion of positive and negative tweets via a 2-step bootstrap method [37]. The first step of the bootstrap sampled individual classifications from the observed data with replacement. The second step labeled the bootstrapped classified data as positive, negative, or neutral based on contingency tables (Multimedia Appendix 1). To compute 2-sided *P* values for the ratio of negative to positive sentiment, we applied a 1-step sample with replacement bootstrap to a null dataset that had the same number of observed neutral tweets, but an equal proportion of positive and negative tweets. The total size of the null dataset matched the observed data, and we assumed classification of the null datasets to be 100% accurate.

### Dissemination of Sentiment

We analyzed word frequency in all original tweets for the most common words in positive and negative tweets. Demographic information about Twitter users was obtained from Demographics Pro (Demographics Pro Inc), a third-party tool providing inferred predictive analytics on demographic information about social media users with 95% or greater confidence based on multiple data sources [26,38,39].

To assess the likelihood of a tweet to be propagated (ie, retweeted), we employed established concepts from the spread of infectious disease [40]. The effective reproduction number equals the expected secondary cases resulting from exposure to an infected individual [41,42]. Analogously, we defined the rate of propagation as the mean number of times a message is retweeted by a Twitter user. To account for heterogeneity of retweeting, we inferred rate of propagation by assigning a negative binomial distribution for the number of retweets each tweet generated. We determined the statistical difference of rate of propagation by Akaike information criterion score [41] and calculated corresponding *P* values by chi-square modeling of the log likelihood ratio. *P* values for the incidence of new tweets were determined based on assuming an underlying Poisson distribution for the introduction of new tweets.

## Results

### Classifier Performance

Our classifier labeled tweet sentiment with an accuracy of about 80%. Importantly, no negatively classified tweets were manually labeled as positive, and only 4% of the positively classified tweets were manually labeled as negative (Multimedia Appendix 1). The misclassifications were predominantly for tweets with nonneutral sentiment classified as being neutral or for tweets with neutral sentiments being classified as nonneutral. As such, the overwhelming majority of misclassified tweets did not entail complete reversal of sentiment. One example of a tweet with neutral sentiment being classified as nonneutral (in this particular case, as negative) is “Worried about preparing for a colonoscopy? Don’t. The preparation can be inconvenient, but it is not difficult or painful.” Since we were using a naive Bayes classification algorithm, the most likely explanation for misclassification of this tweet is the presence of words with negative connotations, such as “painful” and “inconvenient.” Similarly, this nonneutral (in this case, negative) tweet was incorrectly classified as neutral: “cant afford doctor just go to the airport. You get a free xray and breast exam. And if you mention Al Qaeda and you get a free colonoscopy.”

### Differences in Sentiment Among Screening Modalities

**Figure 1:**
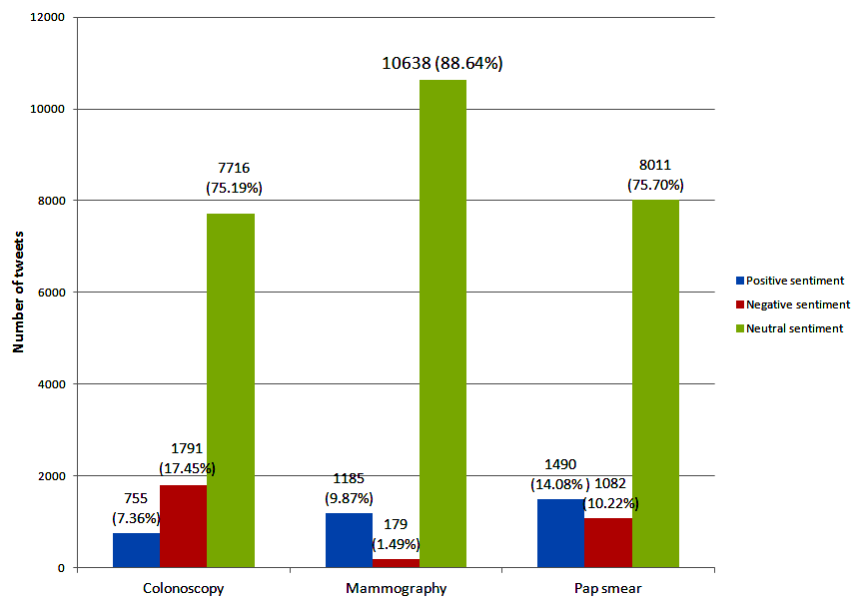
Sentiment expressed on Twitter regarding colonoscopy, mammography, and Pap smear (based on classification of over 30,000 tweets). A naive Bayes classifier was trained on labeled data and used to classify tweets relating to either colonoscopy, mammography, or Pap smear. Tests of statistical significance were undertaken using a bootstrap method with negative to positive sentiment ratio for colonoscopy (1.65, *P*<.001), mammography (0.43, *P*<.001), and Pap smear (0.95, *P*=.18). The full results for the bootstrap analysis are shown in Multimedia Appendix 2.

When adjusted for imperfections in classification, colonoscopy-related tweets were estimated to be 1.65 (95% CI 1.51-1.80, *P*<.001) times more likely to express negative sentiment than positive sentiment (Figure 1 and Multimedia Appendix 2). In contrast, mammography-related tweets were 0.43 (95% CI 0.39-0.47, *P*<.001) times more likely to be negative than positive. The proportions of positive versus negative sentiment in Pap smear-related tweets were not significantly different (negative to positive ratio 0.95, 95% CI 0.87-1.04, *P*=.18). The majority of tweets in all screening modalities were neutral.

### Demographic Analysis

Table 1 provides aggregate sex and age information about Twitter users discussing each screening modality. A large proportion of Twitter users discussing an intervention commonly used for cancer screening were less than 45 years of age, generally younger than those who commonly pursue routine colon cancer screening (typically starting at age 50 years). In contrast to the demographics of the entire Twitter network, which is characterized by roughly equal proportions of male and female users, more male users commented on colonoscopy and, not surprisingly, more female users commented on mammography and Pap smear [43]. Interestingly, Twitter users commenting on colonoscopy, mammography, and Pap smear were younger than the average Twitter user.

**Table 1:** Demographics^a^ of users tweeting about cancer screening by screening modality (N=32,847).

Characteristics		Colonoscopy	Mammography	Pap smear
**Sex**				
	Male	56.7%	36.4%	33.3%
	Female	43.3%	63.6%	66.7%
**Age group (years)**				
	≤20	18.4%	10.9%	24.3%
	21-29	30.8%	20.9%	34.3%
	30-34	15.9%	14.7%	11.8%
	35-44	18.8%	30.7%	18.6%
	45-54	10.1%	15.5%	7.8%
	55-64	4.3%	5.5%	2.2%
	≥65	1.7%	1.5%	1.1%

^a^Percentage data obtained from Demographics Pro.

### Word Frequency Analysis

Word frequency analysis for all 3 screening modalities demonstrated similarly perceived benefits of tools frequently used for cancer screening (Multimedia Appendix 3). Word frequency analysis showed that positively charged tweets most frequently contained words such as “health,” “awareness,” “screening,” “detection,” and “recommend.” Negatively charged tweets most frequently contained words related to pain (“painful,” “hurts”), anxiety associated with the procedure (“worried”), and issues with procedure preparation (“dehydration,” “preparing”). Examination of individual tweets showed that positive sentiment was more likely to be expressed when providing information about a procedure or explaining the benefits of cancer screening (Table 2). Positive tweets might also provide a “call to action” to encourage friends or family to get screened. Negative tweets often expressed physical discomfort related to a screening modality and compared cancer screening to anxiety-provoking tasks or suggested a level of social inappropriateness with the topic. Tweets with negative sentiment often included sarcastic humor.

**Table 2:** Examples of positive and negative tweets.

Type of tweet and modality		Positive sentiment	Negative sentiment	Neutral sentiment
**Information on the procedure (positive) or fear of pain or harm (negative)**				
	Colonoscopy	Worried about preparing for a colonoscopy? Don’t. The preparation can be inconvenient, but it is not difficult or painful.	Getting a colonoscopy can cause the patient to explode (methane+oxygen+electrical spark).	Colonoscopies are not just simple, harmless tests. Here are the pros and cons to consider.
	Mammography	For women with dense breasts, ultrasound could help diagnose breast cancer.	I don’t think my breast have ever been so smashed and squeezed. And here I thought a mammogram would be like taking an X-ray--NOPE! Corsets = struggling to breathe while getting a constant mammogram.	Is This Why Mammogram Recommendations Have Changed?
	Pap smear	Getting a #Paptest is one of the best things you can do to prevent #cervicalcancer.	So my mom never had a pap smear until after she had me, when she was 37. I grew up hearing about how horrific it was. It really hurt her.	For me, a Pap Test just ended up being an unexpected trigger. For various reasons, some of which I will never know.
**Benefits of cancer screening (positive) or “I’d rather” tweets (negative)**				
	Colonoscopy	LOVE your Parents enough to take them in for a Colonoscopy! It could just save their lives! Studies show that the colon cancer death rate was cut by more than half in those who had a colonoscopy. Has your dad turned 50 yet? If so, bug him into getting a colonoscopy. You could be saving his life.	Things I would rather do than my exams: re-organise a forever 21 store, eat my own vomit, peel 4,000 potatoes with a spork, colonoscopy prep I can think of a few better places: Gates of Hell, during a colonoscopy, Mordor, a Joel Osteen Conference...	
	Mammography	Annual mammography in women 40 to 48 y of age reduced breast cancer mortality.	i’d rather have a mammogram done while being awake as they remove my kidney	
	Pap smear	The only way to find changes that may lead cervical cancer is by having a Pap. Screening saves lives!	I would rather give myself a pap smear in the middle of Macy’s than read your Christmas newsletter, Aunt Karen	
**Call to Action (positive) or Other (negative)**				
	Colonoscopy	Have a friend turning 50? Encourage them to get their colonoscopy; it could save their life.	i would hate to get a colonoscopy… My first colonoscopy will be done by a coroner at my autopsy ** **Today a colleague told me that he’s having a colonoscopy this week. I need a new job.	
	Mammography	Ladies get that mammogram because it saved my little sister from a very aggressive breast cancer. Make it a XMas present to yourself.	How a mammogram actually causes breast cancer.	
	Pap smear	I went for my first ever Pap Test today *feeling proud & brave*. Thanks to all the lesbian women who urged/reminded me to go! Hello ladies schedule your mammogram today. Include health in your new year’s resolution.	Your mothers so dumb she went to Dr. Dre for a pap smear	

**Figure 2:**
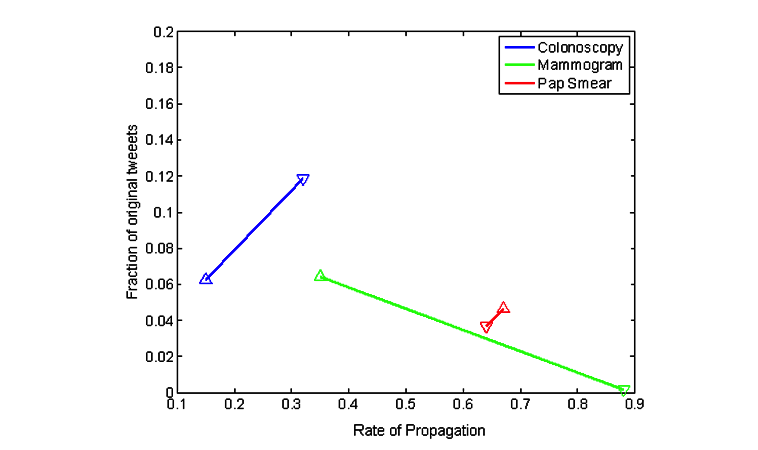
The fraction of original tweets and the rate of propagation for each modality. Upward (downward) pointing triangles represent positive (negative) tweets. Lines are for visualization purposes only. The difference in rate of propagation between positive and negative tweets was significant for colonoscopy (*P*=.001) and mammography (*P*=.02) but not for Pap smear (*P*=.83).

### Propagation of Sentiment

The proportion of tweets with positive versus negative sentiment is dependent on both the frequency of new tweets and the rate at which these new tweets are retweeted (Figure 2 and Multimedia Appendix 4) [44]. Comparison of positively versus negatively classified tweets showed that, for colonoscopy, negative sentiment both was more frequent in original tweets and spread more than positive ones. New tweets regarding mammography were typically positive rather than negative. However, the few negative-sentiment tweets toward mammography spread much more than those with positive sentiment. For Pap smear, negative and positive tweets had similar characteristics with regard to their spread and the frequency of new tweets.

## Discussion

### Principal Results

By using automated sentiment classifiers, we were able to analyze much larger bodies of data than in typical patient survey-based studies. Understanding basic differences in sentiment between interventions commonly used for screening, such as the greater prevalence of negative sentiment regarding colonoscopy compared with mammography, could lead to more targeted, effective interventions, as well as the real-time means to assess the effects of such interventions. Such comparisons could, for example, foster organizations promoting screening to learn from each other to more effectively maintain social media interventions to promote positive sentiment for these lifesaving medical interventions. Beyond sentiment analysis, word frequency analyses can provide quantitative as well as qualitative insight into potential reasons for differences in sentiment and can identify areas on which to focus education efforts. For instance, pain and fear were common themes in negative tweets about all 3 modalities, findings that have been echoed, at times inconsistently, by formal patient surveys [17].

### Comparison With Prior Work

We employed novel machine learning algorithms to understand sentiment on social media regarding tools commonly used for screening. Understanding opinions regarding these changes by analyzing social media could be valuable in assessing health policy changes and implementing new policies. With respect to cancer screening, public reactions to changes in screening recommendations from professional societies could also be monitored, and false perceptions could be addressed immediately.

### Limitations

Despite the large number of public tweets available for analysis, this is an imperfect representation of the population at large. The age of Twitter users sampled is generally younger than the target screening population, who commonly use the medical tests investigated in this study, limiting the generalizability of our results to older populations. However, this limitation also provides useful insight. For example, one hypothesis to explain the negative sentiments regarding colonoscopy in younger users is that some of these persons may have conditions such as inflammatory bowel disease and require invasive, potentially embarrassing interventions that their peers do not require. It is unclear how younger persons’ sentiments toward colonoscopy might affect the sentiments of older persons, including the relatives of the tweet authors, who may be eligible for age-appropriate colon cancer screening. While references to colonoscopy on Twitter may not entirely reflect screening and surveillance, these are the most common indications for colonoscopy [13]. The same is true for mammography and Pap smear. Yet even understanding perceptions of a tool *commonly* used for screening is telling. This information still represents public opinion, albeit a younger population, and understanding the perceptions of this younger population may influence future screening decisions as well. Influencing younger users may in fact be a strategy to further improve cancer screening adherence. The relatively small subgroup sample sizes limited the demographic analysis possible through Demographics Pro. Additionally, potentially vulnerable groups, including minorities at risk for poor preventive health use, may not be represented. Nonetheless, we believe that public expressions of sentiment provide insight that may not necessarily be reflected by formal surveys into how the screening modalities that we studied are perceived by the public. We chose to capture whether tweets mentioning tools commonly used for cancer screening were generally positive or negative versus attempting to discern whether the sentiment was expressed specifically regarding the actual screening procedure itself. More nuanced sentiment analysis methods capable of discerning meaning by analyzing sentences as aggregates of phrases and their modifiers may improve our understanding of public discourse specifically related to cancer screening [45]. We believe that even capturing such nonscreening-related mentions of these interventions provides valuable insight into public opinion for these tools used by millions to improve health.

How well the Twitter API samples the total corpus of tweets (the Twitter Firehose) has been studied by Morstatter et al and has yielded heterogeneous results [32]. The API’s sampling is imperfect and depends to a large extent on the type of analysis undertaken. For example, those authors found that the 1% sampling becomes substantially more representative when tweets are collected over consecutive days, as was the case in our study. The quality of the API’s sampling decreases when the number of hashtags or query keywords decreases, which would theoretically affect the quality of sampled colonoscopy tweets (where “colonoscopy” is the only queried term) more than Pap smear-related tweets (which we sampled using 10 unique keywords). Correcting for this sampling bias is difficult given that Twitter does not disclose how sampling is performed, but it should be acknowledged in infodemiologic studies that use the Twitter search API.

### Conclusion

We have analyzed tweets about interventions commonly used for cancer screening to assess public sentiment about these interventions. There were substantially more negative than positive tweets about colonoscopy, but not mammography or Pap smear. Tweet propagation in the social network was greater for negative than for positive tweets about colonoscopy and mammography, suggesting a possible disproportionate impact of negative sentiment for these screening tests. Examination of large data sets available from the Twitter social network using automated algorithms provides an opportunity to examine public attitudes toward cancer screening and other health care interventions that might lead to policy changes, novel programs, and more refined counseling guidelines that improve public attitudes and health-related behaviors.

## Appendix

Multimedia Appendix 1 Contingency tables for labeling bootstrapped classified data.

Multimedia Appendix 2 Tweet sentiment classification with cancer screen modality.

Multimedia Appendix 3 Word frequency analysis of common themes.

Multimedia Appendix 4 Rate of propagation for positive and negative tweets.

## Abbreviations

API: application programming interface
