# Supplementary material for: Using Social Media to Characterize Public Sentiment Toward Medical Interventions Commonly Used for Cancer Screening: An Observational Study
Source: J Med Internet Res. 2017 Jun 7;19(6):e200. doi: 10.2196/jmir.7485 (PMC5480009; doi:10.2196/jmir.7485)
Supplement: Multimedia Appendix 1 [file jmir_v19i6e200_app1.pdf]

Supplemental Table 1

Contingency tables. The performance of three naive Bayes classifiers was assessed by comparing the sentiment categories assigned by classifiers with human-assigned labels. Sentiment assigned by the classifier is designated as positive (+), negative (-), or neutral (0) and is listed above each 3x3 table. Manually labeled sentiment is provided to the left of each 3x3 contingency table.

		**Colonoscopy**		
		Classifier		
		+	0	-
Labeled	+	5	7	0
	0	1	75	1
	-	0	8	3

		**Pap smear**		
		Classifier		
		+	0	-
Labeled	+	9	3	0
	0	3	74	1
	-	0	4	7

		**Mammography**		
		Classifier		
		+	0	-
Labeled	+	3	21	0
	0	1	56	6
	-	1	9	4
