# Supplementary material for: Using Social Media to Characterize Public Sentiment Toward Medical Interventions Commonly Used for Cancer Screening: An Observational Study
Source: J Med Internet Res. 2017 Jun 7;19(6):e200. doi: 10.2196/jmir.7485 (PMC5480009; doi:10.2196/jmir.7485)
Supplement: Multimedia Appendix 2 [file jmir_v19i6e200_app2.pdf]

Supplemental Table 2

Tweet sentiment classification with cancer screen modality based on classification of over 30,000 tweets. Numbers represent the number of tweets classified as positive, negative, or neutral. The percentages and the negative to positive sentiment ratio for colonoscopy (1.65, 95% CI 1.51-1.80, *P*<.001), mammography (0.43, 95% CI 0.39-0.47, *P*<.001), and Pap smear (0.95, 5% CI 0.87-1.04, *P*=.18) are bootstrapped results that account for classifier error.

	Positive tweets	Negative tweets	Neutral tweets	Negative-positive ratio	Total
Colonoscopy	755 (12.0%: 95% CI 11.2-12.8%)	1791 (19.8%: 95% CI 18.8-20.7%)	7716 (68.2%: 95% CI 67.1-69.4%)	1.65: 95% CI 1.51-1.80	10262
Mammography	1185 (27.6%: 95% CI 26.5-28.7%)	179 (11.9%: 95% CI 11.0-12.7%)	10638 (60.6%: 95% CI 59.4-61.8%)	0.43: 95% CI 0.39-0.47	12002
Pap smear	1490 (13.4%: 95% CI 12.6-14.1%)	1082 (12.7%: 95% CI 11.9-13.4%)	8011 (74.0%: 95% CI 73.0-75.0%)	0.95: 95% CI 0.87-1.04	10583
