# Supplementary material for: Using Social Media to Characterize Public Sentiment Toward Medical Interventions Commonly Used for Cancer Screening: An Observational Study
Source: J Med Internet Res. 2017 Jun 7;19(6):e200. doi: 10.2196/jmir.7485 (PMC5480009; doi:10.2196/jmir.7485)
Supplement: Multimedia Appendix 3 [file jmir_v19i6e200_app3.pdf]

Supplemental Table 3

Word frequency analysis of common themes among positive and negative tweets. Each cell contains the most common words from each category of tweets, in ascending order of frequency. Stop words (such as conjunctions, pronouns, and articles) were excluded.

	Most common positive words	Most common negative words
Colonoscopy	evidence, polyp, fully, scared, prevent, effective, recommend, years, npr*, facts, patients, screened, older, cancer, suites, charges, drops, medicare, screening	dehydration, hours, someone, shit, scheduled, fluids, going, doing, avoid, canal, sotu*, painful, watching, worried, worse, kevinmd*, preparing, secretly, records, patient
Mammography	elderly, screening, extend, suggests, fau, regular, title, benefits, diagnose, dense, breasts, shown, using, comparable, effective, equally, detection, cancer, ultrasound, breast	suffered, pfnet*, emotional, thefosterstv, false, abort, deserve, instead, fuss, yesterday, awful, died, resemble, medical, cancer, defundpp*, machines, butchered, parts, babies
Pap smear	ladies, risk, important, regularly, detection, cervicalhealthmonth, health, vaccine, smears, called, awareness, years, cervicalcancer, prevent, hpv*, regular, screening, tests, cervical, cancer	into, bitches, virgin, mama, lol*, beatbox, sex, shit, means, skip, oregon, california, prescription, pussy, doing, birth, dumb, angela, worried, hurts

*Abbreviations:

NPR = National Public Radio

SOTU = State of the Union

DEFUNDPP = Defund Planned Parenthood

HPV= human papilloma virus

LOL = laugh out loud

PFNET = Pattern-based filter network

KEVINMD = Twitter handle @kevinmd
