# Supplementary material for: Using Social Media to Characterize Public Sentiment Toward Medical Interventions Commonly Used for Cancer Screening: An Observational Study
Source: J Med Internet Res. 2017 Jun 7;19(6):e200. doi: 10.2196/jmir.7485 (PMC5480009; doi:10.2196/jmir.7485)
Supplement: Multimedia Appendix 4 [file jmir_v19i6e200_app4.pdf]

Supplemental Table 4

Rate of propagation for positive and negative tweets among screening modalities. Statistically significant differences between positive and negative tweets are designated with asterisks. ΔAIC scores for colonoscopy, mammography, and Pap smear are 8.2, 3.3, and –2.0 respectively, with corresponding *P* values of .001, .02 and .83.

	Positive sentiment	Negative sentiment	Neutral sentiment
Colonoscopy*	0.15<br>(95% CI 0.10-0.22)	0.32<br>(95% CI 0.25-0.41)	0.26<br>(95% CI 0.24-0.29)
Mammography*	0.35<br>(95% CI 0.28-0.46)	0.88<br>(95% CI 0.54-1.55)	0.23<br>(95% CI 0.21-0.25)
Pap smear	0.67<br>(95% CI 0.58-0.76)	0.64<br>(95% CI 0.50-0.85)	0.33<br>(95% CI 0.30-0.36)
